# Impact of breast cancer subtypes on 3-year survival among adolescent and young adult women

**DOI:** 10.1186/bcr3556

**Published:** 2013-10-16

**Authors:** Theresa H M Keegan, David J Press, Li Tao, Mindy C DeRouen, Allison W Kurian, Christina A Clarke, Scarlett L Gomez

**Affiliations:** 1 Cancer Prevention Institute of California, 2201 Walnut Ave, Suite 300, Fremont, CA 94538, USA; 2 Division of Epidemiology, Department of Health Research and Policy, Stanford University School of Medicine, Stanford, CA 94305-5405, USA; 3 Department of Medicine, Stanford University School of Medicine, 300 Pasteur Drive, Stanford, CA 94305-5110, USA

## Abstract

**Introduction:**

Young women have poorer survival after breast cancer than do older women. It is unclear whether this survival difference relates to the unique distribution of hormone receptor (HR) and human epidermal growth factor receptor 2 (HER2)-defined molecular breast cancer subtypes among adolescent and young adult (AYA) women aged 15 to 39 years. The purpose of our study was to examine associations between breast cancer subtypes and short-term survival in AYA women, as well as to determine whether the distinct molecular subtype distribution among AYA women explains the unfavorable overall breast cancer survival statistics reported for AYA women compared with older women.

**Methods:**

Data for 5,331 AYA breast cancers diagnosed between 2005 and 2009 were obtained from the California Cancer Registry. Survival by subtype (triple-negative; HR+/HER2-; HR+/HER2+; HR-/HER2+) and age-group (AYA versus 40- to 64-year-olds) was analyzed with Cox proportional hazards regression with follow-up through 2010.

**Results:**

With up to 6 years of follow-up and a mean survival time of 3.1 years (SD = 1.5 years), AYA women diagnosed with HR-/HER + and triple-negative breast cancer experienced a 1.6-fold and 2.7-fold increased risk of death, respectively, from all causes (HR-/HER + hazard ratio: 1.55; 95% confidence interval (CI): 1.10 to 2.18; triple-negative HR: 2.75; 95% CI, 2.06 to 3.66) and breast cancer (HR-/HER + hazard ratio: 1.63; 95% CI, 1.12 to 2.36; triple-negative hazard ratio: 2.71; 95% CI, 1.98 to 3.71) than AYA women with HR+/HER2- breast cancer. AYA women who resided in lower socioeconomic status neighborhoods, had public health insurance, and were of Black, compared with White, race/ethnicity experienced worse survival. This race/ethnicity association was attenuated somewhat after adjusting for breast cancer subtypes (hazard ratio, 1.33; 95% CI, 0.98 to 1.82). AYA women had similar all-cause and breast cancer-specific short-term survival as older women for all breast cancer subtypes and across all stages of disease.

**Conclusions:**

Among AYA women with breast cancer, short-term survival varied by breast cancer subtypes, with the distribution of breast cancer subtypes explaining some of the poorer survival observed among Black, compared with White, AYA women. Future studies should consider whether distribution of breast cancer subtypes and other factors, including differential receipt of treatment regimens, influences long-term survival in young compared with older women.

## Introduction

Breast cancer is the most frequently diagnosed cancer among adolescent and young adult (AYA) women between 15 and 39 years of age, accounting for 14% of all AYA cancer diagnoses [1] and 7% of all breast cancer diagnoses [1,2]. Breast cancers are now recognized as heterogeneous, based on tumor expression of receptors for estrogen (ER), progesterone (PR)—referred to jointly as hormone receptor (HR)—and human epidermal growth factor receptor 2 (HER2) [3-5]. We recently reported that AYA breast cancer incidence differs from that in older women, with AYAs having higher proportions of HR+/HER2+, triple-negative, and HR-/HER2+ breast cancer subtypes and higher proportions of patients of non-White race/ethnicity than older women [6]. Compared with older women, AYAs also were more likely to be diagnosed with stage III/IV disease and high-grade tumors [6].

Several recent studies suggest that young age is an independent predictor of poorer survival after breast cancer, even after adjustment for sociodemographic and tumor characteristics [7-14]. The HR+/HER2+ (Luminal B), triple-negative, and HR-/HER2+ subtypes, found in higher proportions among AYAs [6], generally are associated with worse survival than the HR+/HER2- (Luminal A) subtype [15,16], which could explain the overall poor prognosis reported for AYA breast cancer patients. However, only two, small institutionally based studies have examined the extent to which differences in the distributions of breast cancer subtypes explain the lower survival among AYA patients. A 1989 through 2009 hospital-based study in Ireland did not find overall survival differences between AYAs (*n* = 276) and older women when they adjusted for ER, PR, and HER2 status, although HER2 status was only 40% complete, and these three tumor markers were adjusted for separately in the analyses [17]. Conversely, an Italian, institution-based study found worse survival in women <35 years of age (*n* = 315) compared with older women (35 to 50 years of age) for triple-negative, Luminal B, and HER2-positive breast cancer, but not Luminal A breast cancer [14]. To our knowledge, no previous population-based study in the United States has considered survival by molecular breast cancer subtypes among AYAs.

Therefore, by using population-based data from California enriched with subtype information and patient sociodemographic data, we examined associations between breast cancer subtypes and short-term overall and breast cancer-specific survival in AYAs. Additionally, we sought to determine whether the distinct molecular-subtype distribution among AYAs [6] could explain the unfavorable overall breast cancer survival statistics reported for AYAs compared with older women.

## Methods

### Cancer cases

We obtained data from the California Cancer Registry (CCR), which contributes approximately half of the data in the National Cancer Institute’s Surveillance, Epidemiology, and End Results (SEER) program and is estimated to include more than 99% of all invasive cancers diagnosed in California. We included in our analysis all female California residents diagnosed with invasive breast cancer (International Classification of Disease for Oncology, 3^rd^ Edition, (ICD-O-3) site codes C50.0-50.9) during the period January 1, 2005, through December 31, 2009. Ethics approval for human-subjects research was obtained from the California Prevention Institute of California Institutional Review Board. As the analysis was based on state-mandated cancer registry data, the study was conducted in accordance with the waivers of individual informed consent and HIPPA authorization. For each breast cancer case, we obtained cancer registry information routinely abstracted from the medical record [18] on age at diagnosis, race/ethnicity (Hispanic, non-Hispanic White, non-Hispanic Black, and non-Hispanic Asian/Pacific Islander, hereafter referred to as “White”, “Black”, “Hispanic”, and “Asian”), marital status, American Joint Committee on Cancer (AJCC) stage at diagnosis, tumor grade (low (I/II), high (III/IV)), tumor size, lymph node involvement, metastasis status, and ER, PR, and HER2 tumor-expression status. The CCR has collected information on ER and PR since 1990 and on HER2 since 1999 [19]. ER and PR were evaluated with dextran-coated charcoal assays or immunohistochemistry (IHC); HER2 was tested with IHC or fluorescence *in situ* hybridization. The markers were recorded as positive, negative, borderline, not tested, not recorded, or unknown, on the basis of pathology or medical record information at the reporting facility [20].

Before 2005, treatments driving pathologic testing for HER2 were indicated for late-stage breast cancer only; thus data completeness was low, only 59%; since then, data completeness has increased to at least 83%. Because of this data completeness, we limited our analyses to cases diagnosed between 2005 and 2009.

We also obtained registry information on initial course of treatment (surgery, chemotherapy, and radiation therapy), primary source of payment to the hospital at the time of initial diagnosis and/or treatment (health insurance), census-block group of residence at diagnosis, and vital status (routinely determined by the CCR through hospital follow-up and database linkages, including the Social Security Administration) as of December 31, 2010, and, for the deceased, the underlying cause of death.

As information on patient education or other individual-level measures of socioeconomic status (SES) are not collected by the CCR, we assigned a previously developed measure of neighborhood SES based on patient address at time of diagnosis that incorporates 2000 Census block group on education, occupation, unemployment, household income, and poverty [21]. Each cancer case was then assigned to a neighborhood SES quintile based on the distribution of SES across all census block groups in California. Health insurance was grouped into public insurance (Medicaid and other government-assisted programs), private insurance (health maintenance organizations, preferred provider organizations, managed care not otherwise specified, and military care), no insurance, and insurance status unknown [22].

Of the 6,463 California females diagnosed with breast cancer between 15 and 39 years of age and between 2005 and 2009, we excluded cases with *in situ* breast cancer (*n* = 752), Paget disease (*n* < 5), mammographic or xerographic diagnosis only, or no mass found (*n* = 12), breast cancer as a non-first primary (*n* = 364), and autopsy or death certificate only (*n* < 5). The resulting study population included 5,331 AYA patients. For analyses comparing AYAs with older women, we followed the same inclusion criteria, which resulted in 53,860 women from 40 to 64 years of age.

### Categorization of breast cancer subtypes

Breast cancer subtypes were categorized according to tumor expression of ER, PR, and HER2. HR+/HER2- was defined as ER or PR positive and HER2 negative; HR+/HER2+ as ER or PR positive and HER2 positive; HR-/HER2+ as ER and PR negative and HER2 positive; and triple-negative as ER, PR, and HER2 negative [3-5]. Participants who had missing or borderline ER and PR or HER2 and could not be defined in these four categories were considered unclassified (Table 1).

**Table 1:** Demographic and clinical characteristics for adolescents and young adults (15 to 39 years of age) with breast cancer by subtype*, California, 2005 through 2009

	**Total** **(*n*** **= 5,331)**	HR+/HER2-* (*n* **= 2,191)**	HR+/HER2+* (*n* **= 811)**	HR-/HER2+* (*n* **= 469)**	**Triple negative** **(*n*** **= 1014)**	**Unclassified** **(*n*** **= 846)**
Characteristics	*n* (Col%)	*n* (Col%)	*n* (Col%)	*n* (Col%)	*n* (Col%)	*n* (Col%)
Age at diagnosis						
15-29	560 (10.5%)	190 (8.7%)	88 (10.9%)	63 (13.4%)	108 (10.7%)	111 (13.1%)
30-34	1,441 (27.0%)	525 (24.0%)	237 (29.2%)	141 (30.1%)	303 (29.9%)	235 (27.8%)
35-39	3,330 (62.5%)	1476 (67.4%)	486 (59.9%)	265 (56.5%)	603 (59.5%)	500 (59.1%)
Race/ethnicity**						
NH White	2,247 (42.1%)	984 (44.9%)	321 (39.6%)	193 (41.2%)	404 (39.8%)	345 (40.8%)
NH Black	388 (7.3%)	128 (5.8%)	60 (7.4%)	37 (7.9%)	106 (10.5%)	57 (6.7%)
Hispanic	1,737 (32.6%)	645 (29.4%)	273 (33.7%)	163 (34.8%)	378 (37.3%)	278 (32.9%)
NH Asian/Pacific Islander	914 (17.1%)	423 (19.3%)	153 (18.9%)	74 (15.8%)	120 (11.8%)	144 (17.0%)
Other/unknown	45 (0.8%)	11 (0.5%)	<5	<5	6 (0.6%)	22 (2.6%)
Marital status at diagnosis						
Married	3,268 (61.3%)	1,338 (61.1%)	500 (61.7%)	320 (68.2%)	616 (60.7%)	494 (58.4%)
Never married	1,527 (28.6%)	639 (29.2%)	242 (29.8%)	104 (22.2%)	287 (28.3%)	255 (30.1%)
Previously married	380 (7.1%)	165 (7.5%)	56 (6.9%)	31 (6.6%)	83 (8.2%)	45 (5.3%)
Unknown	156 (2.9%)	49 (2.2%)	13 (1.6%)	14 (3.0%)	28 (2.8%)	52 (6.1%)
Tumor grade†						
Low	2,090 (39.2%)	1,288 (58.8%)	323 (39.8%)	102 (21.7%)	91 (9.0%)	286 (33.8%)
High	2,891 (54.2%)	821 (37.5%)	455 (56.1%)	340 (72.5%)	888 (87.6%)	387 (45.7%)
Unknown/not stated	350 (6.6%)	82 (3.7%)	33 (4.1%)	27 (5.8%)	35 (3.5%)	173 (20.4%)
AJCC stage at diagnosis						
I	1,310 (24.6%)	660 (30.1%)	170 (21.0%)	86 (18.3%)	191 (18.8%)	203 (24.0%)
II	2,328 (43.7%)	933 (42.6%)	354 (43.6%)	187 (39.9%)	515 (50.8%)	339 (40.1%)
III	1,100 (20.6%)	425 (19.4%)	214 (26.4%)	130 (27.7%)	208 (20.5%)	123 (14.5%)
IV	313 (5.9%)	115 (5.2%)	47 (5.8%)	47 (10.0%)	60 (5.9%)	44 (5.2%)
Unknown/not stated	280 (5.3%)	58 (2.6%)	26 (3.2%)	19 (4.1%)	40 (3.9%)	137 (16.2%)
Tumor size (cm)						
<2.00	1,987 (37.3%)	969 (44.2%)	309 (38.1%)	147 (31.3%)	287 (28.3%)	275 (32.5%)
2.01-5.00	2,261 (42.4%)	915 (41.8%)	350 (43.2%)	196 (41.8%)	500 (49.3%)	300 (35.5%)
>5.00	734 (13.8%)	239 (10.9%)	115 (14.2%)	79 (16.8%)	176 (17.4%)	125 (14.8%)
Microinvasion	57 (1.1%)	12 (0.5%)	9 (1.1%)	17 (3.6%)	13 (1.3%)	6 (0.7%)
Diffuse	54 (1.0%)	15 (0.7%)	5 (0.6%)	7 (1.5%)	0	27 (3.2%)
Unknown	238 (4.5%)	41 (1.9%)	23 (2.8%)	23 (4.9%)	38 (3.7%)	113 (13.4%)
Lymph nodes involvement						
No	2,563 (48.1%)	1,074 (49.0%)	333 (41.1%)	178 (38.0%)	532 (52.5%)	446 (52.7%)
Positive	2,647 (49.7%)	1,098 (50.1%)	470 (58.0%)	282 (60.1%)	468 (46.2%)	329 (38.9%)
Unknown	121 (2.3%)	19 (0.9%)	8 (1.0%)	9 (1.9%)	14 (1.4%)	71 (8.4%)
Metastasis status						
No	4,880 (91.5%)	2,050 (93.6%)	751 (92.6%)	411 (87.6%)	936 (92.3%)	732 (86.5%)
Yes	316 (5.9%)	115 (5.2%)	47 (5.8%)	47 (10.0%)	61 (6.0%)	46 (5.4%)
Unknown	135 (2.5%)	26 (1.2%)	13 (1.6%)	11 (2.3%)	17 (1.7%)	68 (8.0%)
Surgery						
No	362 (6.8%)	103 (4.7%)	47 (5.8%)	41 (8.7%)	64 (6.3%)	107 (12.6%)
Yes	4,942 (92.7%)	2,077 (94.8%)	763 (94.1%)	428 (91.3%)	944 (93.1%)	730 (86.3%)
Unknown	27 (0.5%)	11 (0.5%)	<5	0	6 (0.6%)	9 (1.1%)
Chemotherapy						
No	1,250 (23.4%)	588 (26.8%)	120 (14.8%)	67 (14.3%)	120 (11.8%)	355 (42.0%)
Yes	3,986 (74.8%)	1,565 (71.4%)	678 (83.6%)	395 (84.2%)	886 (87.4%)	462 (54.6%)
Unknown	95 (1.8%)	38 (1.7%)	13 (1.6%)	7 (1.5%)	8 (0.8%)	29 (3.4%)
Radiation therapy						
No	2,818 (52.9%)	1,110 (50.7%)	415 (51.2%)	244 (52.0%)	503 (49.6%)	546 (64.5%)
Yes	2,509 (47.1%)	1,080 (49.3%)	396 (48.8%)	225 (48.0%)	511 (50.4%)	297 (35.1%)
Unknown	<5	<5	0	0	0	<5
Neighborhood SES quintile						
1, lowest	875 (16.4%)	328 (15.0%)	135 (16.6%)	73 (15.6%)	183 (18.0%)	156 (18.4%)
2	997 (18.7%)	392 (17.9%)	133 (16.4%)	97 (20.7%)	203 (20.0%)	172 (20.3%)
3	1,051 (19.7%)	411 (18.8%)	163 (20.1%)	103 (22.0%)	197 (19.4%)	177 (20.9%)
4	1,190 (22.3%)	524 (23.9%)	178 (21.9%)	100 (21.3%)	206 (20.3%)	182 (21.5%)
5, highest	1,218 (22.8%)	536 (24.5%)	202 (24.9%)	96 (20.5%)	225 (22.2%)	159 (18.8%)
Insurance status‡						
Private/military insurance	3,637 (68.2%)	1564 (71.4%)	557 (68.7%)	310 (66.1%)	680 (67.1%)	526 (62.2%)
Public insurance	1,109 (20.8%)	408 (18.6%)	175 (21.6%)	109 (23.2%)	234 (23.1%)	183 (21.6%)
No insurance	93 (1.7%)	30 (1.4%)	16 (2.0%)	6 (1.3%)	13 (1.3%)	28 (3.3%)
Unknown	492 (9.2%)	189 (8.6%)	63 (7.8%)	44 (9.4%)	87 (8.6%)	109 (12.9%)

* Human epidermal growth factor receptor 2 (HER2), hormone receptor (HR), triple-negative (estrogen-receptor negative, progesterone-receptor negative, HER2-negative).

** Non-Hispanic.

† Low grade was defined as tumor grade I and II; high grade was defined as tumor grade III.

‡ Public insurance included Medicaid and other government-assisted programs; private insurance included health maintenance organizations, preferred provider organizations, managed care not otherwise specified, and military care.

### Statistical analyses

To evaluate differences in survival by breast cancer subtypes, we conducted survival analyses with Cox proportional hazards regression to estimate hazard ratios and associated 95% confidence intervals (95% CIs). For deceased patients, survival time was measured in days from the date of diagnosis to the date of death from any cause for overall survival or to the date of death from breast cancer for breast cancer-specific survival. Patients who died of other causes were censored at the time of death for analyses of breast cancer-specific survival. Patients alive at the study end date (December 31, 2010) were censored at this time or at date of last follow-up (that is, last known contact); 96% of censored patients had a follow-up date within 2 years of the study end date.

The proportional hazards assumption was examined by statistical testing of the correlation between weighted Schoenfeld residuals and logarithmically transformed survival time. No violations of the assumption were observed. Multivariate Cox regression models included cancer registry variables significant at *P* < 0.05 in univariate models (age at diagnosis, subtype, race/ethnicity, marital status, tumor grade, lymph node involvement, tumor size, neighborhood SES, health insurance status, surgery, and radiation therapy) or with *a priori* hypotheses for inclusion (for example, chemotherapy). Models were conducted with and without breast cancer subtype, and AJCC stage was included as a stratifying variable. Effect modification between breast cancer subtypes and race/ethnicity, tumor grade, lymph node involvement, neighborhood SES, and health insurance status was assessed by including an interaction term in the multivariable model; a significant interaction (*P* < 0.05) was found between subtype and tumor grade. Hazard ratio and 95% CI estimates for comparing AYAs with women 40 to 64 years of age were presented by stage of diagnosis for comparison with prior studies [1,8]. Analyses were carried out by using SAS software version 9.3 (SAS Institute, Cary, NC, USA). All *P* values reported are two-sided, and those that were <0.05 were considered to be statistically significant.

## Results

HR+/HER2- was the most commonly diagnosed subtype (41.1%) among AYA breast cancer patients, followed by triple-negative (19.0%), HR+/HER2+ (15.2%), and HR-/HER2+ (8.8%) (Table 1). In this California cohort, most AYA patients were of White (42.1%) or Hispanic (32.6%) race/ethnicity; 62.5% of AYAs were diagnosed between 35 and 39 years of age. The highest proportion of stage III/IV disease occurred for the HR+/HER2+ and HR-/HER2+ subtypes, and the highest proportion of high-grade disease occurred for the HR-/HER2+ and triple-negative subtypes. The proportion of AYA patients who received chemotherapy ranged from 71.4% for HR+/HER2- to 87.4% for triple-negative subtypes; the proportions of AYAs who received surgery or radiation were similar across subtypes.

**Figure 1:**
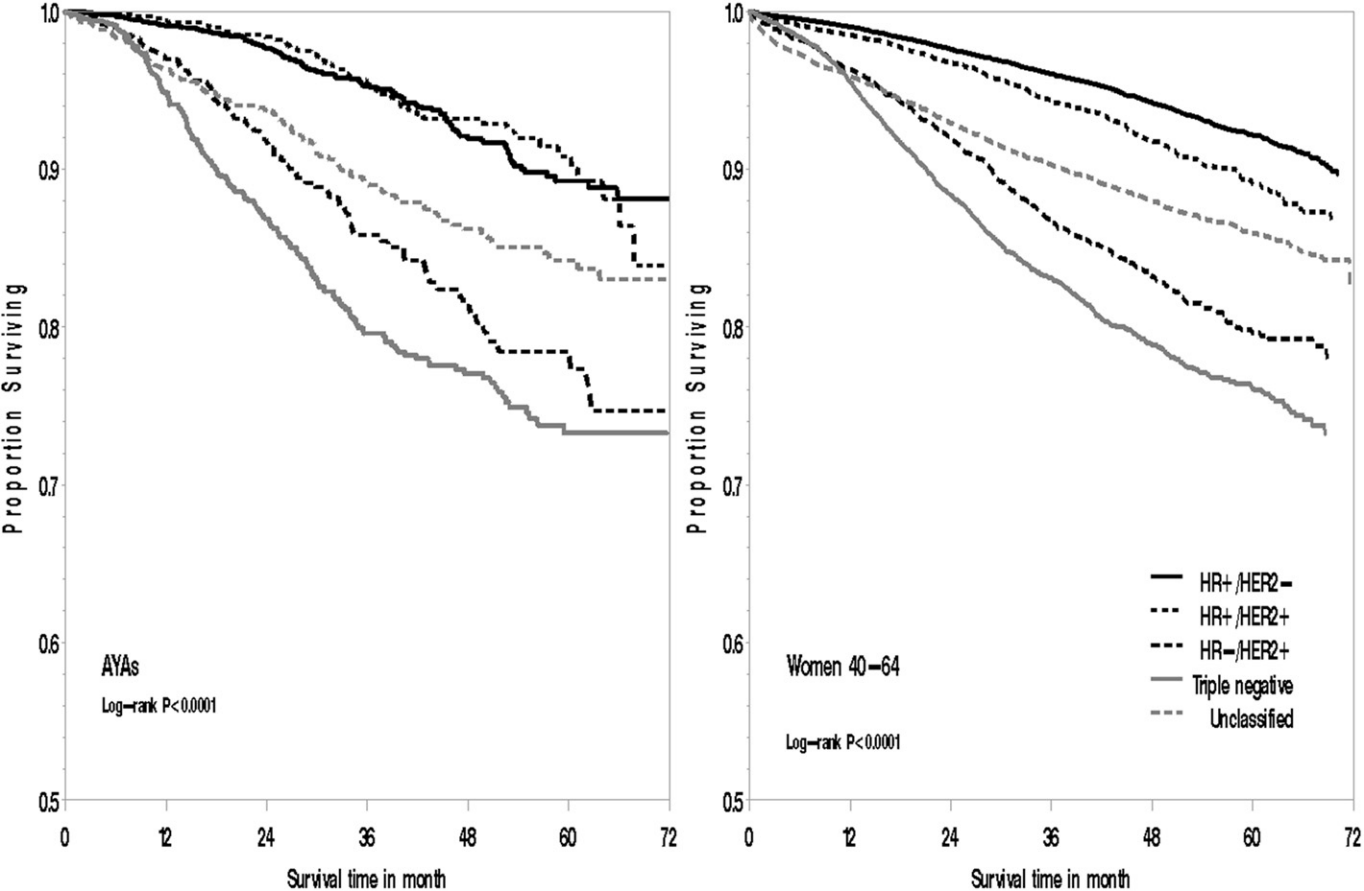
Overall survival for adolescents and young adults (15 to 39 years of age) and women aged 40 to 64 years with breast cancer by subtype, California, 2005 through 2009.

With up to 6 years of follow-up and a mean survival time of 3.1 years (SD = 1.5 years), AYAs diagnosed with HR-/HER + and triple-negative breast cancer experienced an approximately 1.6-fold and 2.7-fold increased risk of death, respectively, from all causes (HR-/HER+ hazard ratio: 1.55; 95% CI, 1.10 to 2.18; triple-negative HR: 2.75; 95% CI, 2.06 to 3.66) and breast cancer (HR-/HER+ hazard ratio: 1.63; 95% CI, 1.12 to 2.36; triple-negative hazard ratio: 2.71; 95% CI, 1.98 to 3.71) compared with AYAs diagnosed with HR+/HER2-; however, among AYAs, survival was similar between HR+/HER2- and HR+/HER2+ breast cancer subtypes (Figure 1, Table 2). Adjusting for breast cancer subtypes attenuated the poorer survival experienced by Black, compared with White, AYAs. Although no longer statistically significant, Black AYAs had a 33% increased risk of death over Whites after adjustment for breast cancer subtype (hazard ratio, 1.33; 95% CI, 0.98 to 1.82). AYA patients residing in lower-SES neighborhoods generally had poorer survival than did AYAs residing in the highest SES neighborhood quintile, although not all of these associations remained statistically significant after stratifying by stage at diagnosis and adjusting for health insurance and treatment. In particular, in models without stage at diagnosis, health insurance, and treatment, an 86% increased risk of death was found from all causes (hazard ratio, 1.86; 95% CI, 1.32 to 2.61) and an 80% increased risk of death of breast cancer (hazard ratio, 1.80; 95% CI, 1.24 to 2.63) among AYAs residing in the lowest versus highest SES neighborhoods (data not shown in tables). In addition, AYA patients with public, compared with private, medical insurance also experienced worse survival.

**Table 2:** Risk of death from any cause or breast cancer among adolescents and young adults (15 to 39 years of age) with breast cancer*, California, 2005 through 2009

	**All-cause deaths**			**Breast cancer-specific deaths**		
	**Number of deaths**	Model 1* HR (95% CI)	Model 2** HR (95% CI)	**Number of deaths**	Model 1* HR (95% CI)	Model 2** † **HR (95% CI)**
Subtype†						
HR+/HER2-	124	N/A	1.00 (reference)	100	N/A	1.00 (reference)
HR+/HER2+	46		0.77 (0.53-1.11)	40		0.80 (0.54-1.19)
HR-/HER2+	73		1.55 (1.10-2.18)	66		1.63 (1.12-2.36)
Triple negative	194		2.75 (2.06-3.66)	166		2.71 (1.98-3.71)
Unclassified	97		1.34 (0.96-1.86)	82		1.37 (0.95-1.98)
Race/ethnicity‡						
NH White	194	1.00 (reference)	1.00 (reference)	164	1.00 (reference)	1.00 (reference)
NH Black	77	1.55 (1.14-2.10)	1.33 (0.98-1.82)	67	1.57 (1.12-2.20)	1.35 (0.96-1.89)
Hispanic	199	1.08 (0.85-1.38)	1.06 (0.83-1.35)	172	1.15 (0.88-1.49)	1.12 (0.86-1.46)
NH Asian/Pacific Islander	61	0.99 (0.73-1.35)	1.00 (0.73-1.36)	48	0.98 (0.69-1.39)	0.98 (0.69-1.39)
Unknown	<5	N/A	N/A	<5	N/A	N/A
Marital status at diagnosis						
Married	284	1.00 (reference)	1.00 (reference)	241	1.00 (reference)	1.00 (reference)
Never married	190	1.26 (1.01-1.58)	1.26 (1.01-1.57)	163	1.29 (1.01-1.64)	1.28 (1.01-1.63)
Previously married	41	1.18 (0.81-1.70)	1.09 (0.75-1.57)	35	1.17 (0.78-1.77)	1.08 (0.72-1.63)
Unknown	19	1.26 (0.74-2.15)	1.15 (0.67-1.95)	15	1.17 (0.64-2.15)	1.05 (0.58-1.93)
Tumor grade						
Low	113	1.00 (reference)	1.00 (reference)	84	1.00 (reference)	1.00 (reference)
High	374	1.92 (1.52-2.43)	1.50 (1.17-1.93)	332	2.30 (1.76-3.01)	1.81 (1.36-2.41)
Unknown	47	1.01 (0.60-1.71)	0.84 (0.49-1.44)	38	1.12 (0.61-2.04)	0.92 (0.50-1.70)
Lymph nodes involvement						
No	124	1.00 (reference)	1.00 (reference)	98	1.00 (reference)	1.00 (reference)
Positive	372	1.15 (0.87-1.52)	1.23 (0.93-1.62)	321	1.16 (0.85-1.58)	1.24 (0.91-1.70)
Unknown	38	0.86 (0.44-1.69)	0.84 (0.43-1.65)	35	1.11 (0.54-2.28)	1.10 (0.53-2.26)
Neighborhood SES quintile						
5 (highest)	73	1.00 (reference)	1.00 (reference)	60	1.00 (reference)	1.00 (reference)
4	103	1.21 (0.87-1.68)	1.30 (0.93-1.80)	90	1.25 (0.88-1.79)	1.33 (0.93-1.90)
3	111	1.35 (0.97-1.87)	1.41 (1.01-1.95)	92	1.27 (0.88-1.82)	1.31 (0.91-1.88)
2	117	1.35 (0.97-1.88)	1.42 (1.02-1.98)	101	1.37 (0.96-1.96)	1.43 (1.00-2.06)
1 (lowest)	130	1.19 (0.84-1.70)	1.23 (0.86-1.75)	111	1.13 (0.77-1.66)	1.15 (0.78-1.70)
Insurance status^×^						
Private/military insurance	272	1.00 (reference)	1.00 (reference)	222	1.00 (reference)	1.00 (reference)
Public insurance	192	1.53 (1.21-1.92)	1.52 (1.21-1.92)	171	1.64 (1.28-2.10)	1.63 (1.27-2.10)
No insurance	14	0.95 (0.49-1.83)	1.08 (0.56-2.09)	12	0.92 (0.44-1.92)	1.04 (0.50-2.17)
Unknown	56	1.33 (0.94-1.88)	1.33 (0.95-1.88)	49	1.48 (1.01-2.15)	1.45 (1.00-2.11)

* Cox models were adjusted for all variables in the table and age at diagnosis (continuous), tumor size (continuous), and first course of treatment (chemotherapy (y/n), radiation (y/n), and surgery (y/n)); AJCC stage levels I through IV and unknown was included as a stratifying variable.

** Cox models were additionally adjusted for subtypes of breast cancer.

† Human epidermal growth factor receptor 2 (HER2), hormone receptor (HR), triple-negative (estrogen-receptor negative, progesterone-receptor negative, HER2-negative).

‡ Non-Hispanic.

^×^ Public insurance included Medicaid and other government-assisted programs; private insurance included health maintenance organizations, preferred provider organizations, managed care not otherwise specified, and military care.

We observed an interaction (*P v*alue = 0.02 for overall survival and *P* value = 0.01 for breast cancer-specific survival) between breast cancer subtype and tumor grade (Table 3). Among AYA patients diagnosed with low-grade tumors, the risk of breast cancer death was nearly 14 times higher in women with triple-negative and 2 times higher in women with HR+/HER2+ compared with HR+/HER2- breast cancer. For high-grade tumors, AYAs diagnosed with triple-negative breast cancer had more than two-fold increased risk of death, whereas AYAs diagnosed with HR+/HER2+ breast cancer experienced approximately half the risk of death, than did AYAs diagnosed with HR+/HER2- breast cancer.

**Table 3:** Risk of death of any cause or of breast cancer for adolescents and young adults (15 to 39 years of age) with breast cancer by tumor grade*, California, 2005-2009

	**All-cause deaths**		**Breast cancer-specific deaths**	
	**Number of deaths**	HR (95% CI)*	**Number of deaths**	HR (95% CI)*
Low tumor grade (I/II)				
Subtype†				
HR+/HER2-	44	1.00 (reference)	27	1.00 (reference)
HR+/HER2+	19	1.57 (0.87-2.81)	16	2.23 (1.12-4.44)
HR-/HER2+	9	1.52 (0.61-3.76)	7	2.37 (0.85-6.61)
Triple negative	25	10.64 (5.66-20.00)	21	13.87 (6.49-29.64)
Unclassified	16	1.34 (0.70-2.57)	13	2.05 (0.95-4.44)
High tumor grade (III)				
Subtype†				
HR+/HER2-	73	1.00 (reference)	67	1.00 (reference)
HR+/HER2+	23	0.49 (0.29-0.81)	21	0.48 (0.28-0.81)
HR-/HER2+	59	1.40 (0.95-2.06)	55	1.40 (0.93-2.10)
Triple negative	162	2.24 (1.61-3.13)	140	2.14 (1.51-3.04)
Unclassified	57	1.47 (0.98-2.21)	49	1.32 (0.85-2.05)

* Cox models were adjusted for all variables presented in Table 2 and age at diagnosis (continuous), tumor size (continuous), and first course of treatment (chemotherapy (y/n), radiation (y/n), and surgery (y/n)); AJCC stage levels I-IV and unknown was included as a stratifying variable. P for interaction between breast cancer subtype and grade = 0.018 for all-cause mortality and =0.0735 for breast cancer-specific mortality.

† Human epidermal growth factor receptor 2 (HER2), hormone receptor (HR), triple-negative (estrogen-receptor negative, progesterone-receptor negative, HER2-negative).

Overall, in models including only age group, AYA patients experienced a 30% increased risk of death from all causes (hazard ratio, 1.30; 95% CI, 1.19 to 1.42) and 44% increased risk of death from breast cancer (hazard ratio, 1.44; 95% CI, 1.30 to 1.59) than women 40 to 64 years of age (data not shown in tables). Once models were stratified by stage at diagnosis, AYAs had similar short-term all-cause (hazard ratio, 0.99; 95% CI, 0.90 to 1.08) and breast cancer-specific survival (hazard ratio, 1.05; 95% CI, 0.96 to 1.16) to women 40 to 64 years of age (data not shown in tables). Further adjustment for other factors and breast cancer subtype did not appreciably change these associations (all-cause survival hazard ratio, 0.92; 95% CI, 0.83 to 1.02; breast cancer-specific survival hazard ratio, 0.94; 95% CI, 0.84 to 1.05) (Table 4). AYAs demonstrated similar survival to older women for all breast cancer subtypes, across all race/ethnicities, and across all stages of disease (Table 4). Although not statistically significant, AYAs with early stage I disease may have poorer all-cause (hazard ratio, 1.44; 95% CI, 0.90 to 2.31) and breast cancer-specific survival (hazard ratio, 1.38; 95% CI, 0.86 to 2.21) than women 40 to 64 years of age.

**Table 4:** Risk of death of any cause or of breast cancer, comparing younger women (15 to 39 years of age) with women 40 to 64 years of age with breast cancer, by subtype, race/ethnicity, or stage at diagnosis; California, 2005 through 2009

	**All-cause deaths**		**Breast cancer-specific deaths**	
	Model 1* HR (95% CI)	Model 2** HR (95% CI)	Model 1** HR (95% CI)	Model 2** HR (95% CI)
Overall	0.96 (0.86-1.06)	0.92 (0.83-1.02)	0.99 (0.88-1.10)	0.94 (0.84-1.05)
Subtype†				
HR+/HER2-	N/A	0.92 (0.75-1.11)	N/A	0.92 (0.74-1.15)
HR+/HER2+		0.87 (0.62-1.22)		1.00 (0.69-1.46)
HR-/HER2+		0.99 (0.74-1.31)		1.08 (0.80-1.46)
Triple negative		0.93 (0.78-1.10)		0.93 (0.77-1.12)
Unclassified		0.86 (0.66-1.13)		0.88 (0.65-1.19)
Race/ethnicity‡				
NH White	0.90 (0.76-1.06)	0.89 (0.75-1.05)	0.93 (0.78-1.12)	0.92 (0.77-1.11)
NH Black	0.93 (0.71-1.21)	0.89 (0.68-1.16)	0.97 (0.72-1.29)	0.91 (0.68-1.22)
Hispanic	1.01 (0.85-1.20)	0.95 (0.80-1.14)	1.05 (0.87-1.27)	0.99 (0.82-1.20)
NH Asian/Pacific Islander	1.01 (0.75-1.35)	0.98 (0.73-1.31)	1.02 (0.73-1.41)	0.98 (0.71-1.37)
Unknown	N/A	N/A	N/A	N/A
AJCC stage at diagnosis				
I	0.97 (0.65-1.43)	0.95 (0.64-1.40)	1.44 (0.90-2.31)	1.38 (0.86-2.21)
II	0.95 (0.79-1.15)	0.90 (0.74-1.08)	0.98 (0.79-1.21)	0.91 (0.73-1.13)
III	1.02 (0.86-1.21)	0.97 (0.82-1.15)	1.06 (0.88-1.27)	1.01 (0.84-1.21)
IV	0.86 (0.70-1.06)	0.86 (0.70-1.05)	0.86 (0.69-1.07)	0.86 (0.69-1.07)
Unknown	1.10 (0.70-1.74)	1.09 (0.69-1.72)	1.02 (0.59-1.77)	1.03 (0.59-1.78)

* Reference group for each model was women 40 to 64 years of age at diagnosis. Cox models were adjusted for all variables in the table and year of diagnosis (continuous), marital status at diagnosis, tumor grade, neighborhood SES (quintile), and first course of treatment (chemotherapy (y/n), radiation (y/n), and surgery (y/n)). AJCC stage levels I through IV and unknown were included as a stratifying variable.

** Cox models were additionally adjusted for subtypes of breast cancer.

† Human epidermal growth factor receptor 2 (HER2), hormone receptor (HR), triple-negative (estrogen-receptor negative, progesterone-receptor negative, HER2-negative).

‡ Non-Hispanic.

## Discussion

Using recently available data on HER2-defined breast cancer in the large, diverse California population, to our knowledge, our study is the first to conduct a population-based assessment of subtype-specific breast cancer survival among AYA patients. AYAs diagnosed with HR-/HER2+ and triple-negative breast cancer experienced poorer overall and breast cancer-specific survival than did AYAs diagnosed with HR+/HER2- breast cancer. For AYAs diagnosed with HR+/HER2+ breast cancer, prognosis varied by tumor grade, with the risk of breast cancer death higher in low-grade disease and lower in high-grade disease than AYAs diagnosed with HR+/HER2- breast cancer. In addition, poorer survival outcomes were observed among AYAs who resided in lower-SES neighborhoods, had public health insurance, and who were of Black, compared with White, race/ethnicity, although the race/ethnicity association was attenuated somewhat after adjusting for breast cancer subtypes. Furthermore, across all breast cancer subtypes and after consideration for stage at diagnosis and other factors, AYA women experienced similar short-term survival compared with older women.

Prior studies reported younger age at diagnosis as an independent predictor of adverse prognosis [7,9-11,13,14]. Whereas our study found similar short-term survival among AYAs and older women overall and for all breast cancer subtypes, an institution-based study in Italy found poorer survival in women <35 years for all subtypes, except Luminal A breast cancer [14]. The poorer survival among AYAs has been attributed to the lack of routine screening among women younger than 40 years, whose diagnoses tend to follow identification of a palpable mass and concomitant differences in clinical presentation [23] or differential receipt of treatment regimens previously observed between AYAs and older age groups [11,24,25]. In particular, uncertainty exists regarding the optimal endocrine therapy for premenopausal women, with some clinical trials suggesting that more intensive therapy, including ovarian suppression or ablation, improves outcomes [26,27]. Despite the disproportionate burden of advanced-stage disease at diagnosis among AYA women [6], worse survival among AYA women has been attributed to poorer outcomes with early-stage disease [8,10,11], a finding suggested by our study. Overall, our findings suggest that differences in stage at diagnosis between AYAs and older women [6] explain the poorer short-term survival observed in young women.

AYA breast cancer patients of Black race/ethnicity experienced worse short-term survival than did White women of the same age. Poorer survival outcomes have been reported among young Black women, who also experience a disproportionate burden of triple-negative breast cancer, compared with young White women [6,8,13,28]. Among AYAs, we previously reported that, relative to Whites, Blacks were diagnosed with a higher proportion of triple-negative breast cancer [6]. Although adjusting for breast cancer subtype attenuated survival disparities somewhat, Black AYAs still experienced poorer survival than White AYAs. Black AYA and older women experienced similar breast cancer-specific survival, underscoring the poorer survival of black women seen at all ages [29].

Our findings of poorer survival among AYA breast cancer patients living in lower SES neighborhoods is supported by previous studies involving women of all ages using SEER data [30-32]. Although explanations for SES differences in survival are not well documented, advanced stage at diagnosis has been the most cited explanatory factor [32,33]—perhaps because of screening disparities among women age 40 to 79 years [34]—and increasing evidence suggests inadequate breast cancer treatment and follow-up care among patients in lower SES groups [33]. Furthermore, recent evidence suggests that disparities in breast cancer treatment modalities are associated with health insurance status [35-37], a measure we found to be associated with survival in our study. Factors related to treatment receipt and stage at diagnosis, however, have not been found to explain fully the socioeconomic disparities in survival [32,33,38,39]. Our study observed neighborhood SES differences when we controlled for stage at diagnosis, initial course of treatment, and health insurance, although controlling for these three factors attenuated our neighborhood SES findings, particularly for the lowest SES neighborhoods. SES inequalities in survival also may be influenced by factors we could not measure in this study, such as quality of treatment and follow-up care, and comorbidities [33].

Among AYAs, worse short-term survival for HR-/HER2+ and triple-negative breast cancers is consistent with prior studies that had 5 or fewer years of follow up [14,40,41]. Studies with more than 5 years of follow-up have demonstrated that triple-negative breast cancer may not have the worst long-term survival outcomes among subtypes [16,42-45], perhaps because of an early peak of recurrence in the first few years after diagnosis and a sharp decrease in the recurrence rate in subsequent years [46]. Results from the population-based Carolina Breast Cancer Study demonstrated that the risk of death 10 or more years after diagnosis was highest among ER-/HER2+ breast cancers for both White and Black women [16,42]. Our finding of an interaction between breast cancer subtype and tumor grade suggests that low-grade triple-negative breast cancers, which are less common, have substantially worse prognosis than low-grade HR+/HER2- breast cancer. Furthermore, the higher risk of breast cancer death in low-grade HR+/HER2+ disease and lower risk of death in high-grade HR+/HER2+ disease than HR+/HER2- disease could result from women with high-grade HR+/HER2+ disease being more likely than those with low-grade HR+/HER2+ disease to receive adjuvant trastuzumab, as suggested by recent studies on patterns of care [47-49].

Our study is the first, to our knowledge, to use population-based cancer registry data to examine survival by the four major molecular breast cancer subtypes for AYA patients by race/ethnicity. As compared with prior studies, ours had a relatively low percentage (16%) of women whose breast cancers were unclassified because of missing ER, PR, or HER2 receptor information [19,50]. To maximize the availability of HER2-receptor status, however, our study was restricted to diagnoses between 2005 and 2009, limiting the number of AYAs included and the survival time available. Although reliability of ER and PR tests can be controversial [51], evidence suggests that results from a centralized pathology laboratory generally agree with registry reports for ER and PR status [52]. However, HER2 testing between community-based hospitals and centralized reference laboratories has been found to contain some disagreement [53]. Consensus-based methods to improve laboratory assays will continue to increase the reliability of ER, PR, and HER2 tests over time [54,55]. As with other studies that consider breast cancer subtype according to receptor status, we may be limited, in that subtypes determined by ER, PR, and HER2 receptor status serve only as a proxy for full genetic profiling. These ER, PR, and HER2 designations, however, guide clinical treatment [56] and are becoming increasingly useful in epidemiologic research [3,16,57]. Our study is also subject to the potential misclassification of race/ethnicity, although we have detected excellent overall agreement with self-reported race/ethnicity for Whites and Blacks, and good agreement for Hispanics and Asians [58,59]. Although we considered the first course of cancer-directed treatment, we did not have details on treatment such as chemotherapy components and regimen, or treatment received after this period; therefore, our findings could be subject to residual confounding from incomplete treatment data in the cancer registry [60]. We also lacked information about treatment failure or recurrence. Furthermore, our study did not have individual-level measures of SES to consider separately or with our neighborhood measure. Although neighborhood and individual SES are associated, neighborhood SES has been found to underestimate associations observed with individual-level SES [61].

## Conclusions

Among AYAs with breast cancer, short-term survival varied by breast cancer subtypes, with AYAs diagnosed with HR-/HER2+ and triple-negative breast cancer experiencing poorer short-term survival than AYAs diagnosed with HR+/HER2- breast cancer. In addition, poorer survival outcomes were observed among AYAs who resided in lower SES neighborhoods, had public health insurance, and who were of Black, compared with White, race/ethnicity. Although differences in the breast cancer subtype distributions explained some of the worse survival observed for Black compared with White AYAs, it is noteworthy that AYA and older Black women had similar breast cancer survival, underscoring the poorer survival among Black women at all ages. As follow-up data accrue, it will be important to continue to monitor long-term survival by breast cancer subtypes in the AYA population. We did not observe short-term survival differences between AYAs and older women after consideration for stage at diagnosis, but future studies should consider whether the distribution of breast cancer subtypes and other factors, including differential receipt of treatment regimens, influences long-term survival in young compared with older women.

## Abbreviations

AJCC: American Joint Committee on Cancer; AYA: Adolescent and young adult; CCR: California Cancer Registry; CI: Confidence interval; ER: Estrogen receptor; HER2: Human epidermal growth factor receptor 2; HR: Hormone receptor; IHC: Immunohistochemistry; PR: Progesterone receptor; SEER: Surveillance, Epidemiology, and End Results; SES: Socioeconomic status.

## Competing interests

The authors have no competing interest to disclose.

## Authors’ contributions

DJP and LT performed the statistical analyses. DJP, LT, MCD, AWK, CAC, and SLG participated in the interpretation of data and drafting and critical review of the manuscript. THMK designed the study, interpreted the data, and led the writing and review of the manuscript. All authors read and approved the final manuscript.
